# Investigating the role of mGluR2 versus mGluR3 in antipsychotic-like effects, sleep-wake architecture and network oscillatory activity using novel Han Wistar rats lacking mGluR2 expression

**DOI:** 10.1016/j.neuropharm.2018.07.013

**Published:** 2018-09-15

**Authors:** Christian M. Wood, Keith A. Wafford, Andrew P. McCarthy, Nicola Hewes, Elaine Shanks, David Lodge, Emma S.J. Robinson

**Affiliations:** a School of Physiology, Pharmacology and Neuroscience, University of Bristol, University Walk, Bristol, BS8 1TD, United Kingdom; b Neuroscience Division, Eli Lilly & Co. Ltd., Windlesham, GU20 6PH, United Kingdom; c Department of Physiology, Development and Neuroscience, University of Cambridge, Cambridge, United Kingdom

**Keywords:** Metabotropic glutamate receptor, mGluR2, mGluR3, Han Wistar, Hyperlocomotion, Sleep, EEG, EEG, electroencephalography, mGluR2, metabotropic glutamate receptor 2, mGluR3, metabotropic glutamate receptor 3, HFO, high frequency oscillations, Grm2, glutamate receptor metabotropic 2

## Abstract

Group II metabotropic glutamate receptors (mGluR2 and mGluR3) are implicated in a number of psychiatric disorders. They also control sleep-wake architecture and may offer novel therapeutic targets. However, the roles of the mGluR2 versus mGluR3 subtypes are not well understood. Here, we have taken advantage of the recently described mutant strain of Han Wistar rats, which do not express mGluR2 receptors, to investigate behavioural, sleep and EEG responses to mGluR2/3 ligands.

The mGluR2/3 agonist, LY354740 (10 mg/kg), reversed amphetamine- and phencyclidine-induced locomotion and rearing behaviours in control Wistar but not in mGluR2 lacking Han Wistar rats. In control Wistar but not in Han Wistar rats the mGluR2/3 agonist LY379268 (3 & 10 mg/kg) induced REM sleep suppression with dose-dependent effects on wake and NREM sleep. By contrast, the mGluR2/3 antagonist LY3020371 (3 & 10 mg/kg) had wake-promoting effects in both rat strains, albeit smaller in the mGluR2-lacking Han Wistar rats, indicating both mGluR2 and mGluR3-mediated effects on wakefulness. LY3020371 enhanced wake cortical oscillations in the theta (4–9 Hz) and gamma (30–80 Hz) range in both Wistar and Han Wistar rat strains, whereas LY379268 reduced theta and gamma oscillations in control Wistar rats, with minimal effects in Han Wistar rats.

Together these studies illustrate the significant contribution of mGluR2 to the antipsychotic-like, sleep and EEG effects of drugs acting on group II mGluRs. However, we also provide evidence of a role for mGluR3 activity in the control of sleep and wake cortical theta and gamma oscillations.

## 1 Introduction

Modulation of glutamate neurotransmission through group II metabotropic glutamate receptors (mGluR2 and mGluR3) is an area of promise for the treatment of psychiatric and neurological disorders (Niswender and Conn, 2010; Nicoletti et al., 2011; Chaki et al., 2013; Li et al., 2015). These receptors are primarily considered as auto- and hetero-receptors that reduce the release of neurotransmitters such as glutamate and GABA (Di Iorio et al., 1996; Jones et al., 1998; Cartmell and Schoepp, 2000; Smolders et al., 2004). These actions occur through inhibitory Gα_i/o_ signaling that reduces cAMP formation and modulates pathways such as PKA and ERK/MAPK (Tanabe et al., 1992; Pin and Duvoisin, 1995; Harris et al., 2004). Effects mediated through Gβ/γ proteins modulate exocytosis through inhibition of calcium channels and activation of potassium channels (Chavis et al., 1994; Knoflach and Kemp, 1998). Furthermore, mGluR2/3 can also mediate changes in excitatory and inhibitory neurotransmission through altering NMDA and AMPA receptor function (Tyszkiewicz et al., 2004; Xi et al., 2011; Wang et al., 2013).

Involvement of glutamate in the pathophysiology of schizophrenia is an established hypothesis (Olney and Farber, 1995; Tamminga, 1998; Carlsson et al., 2001; Coyle and Tsai, 2004; Javitt, 2010) with aberrant glutamatergic neurotransmission suggested to be central to the disorder (Javitt and Zukin, 1991). Preclinical work has shown that mGluR2/3 receptor agonists have antipsychotic-like effects in a number of behavioural models (Moghaddam and Adams, 1998; Cartmell et al., 2000; Fell et al., 2008; Jones et al., 2011; but also see Cartmell et al., 1999) with data in knockout mice suggesting mGluR2 are critical for these effects (Spooren et al., 2000; Johnson et al., 2005; Rorick-Kehn et al., 2007; Woolley et al., 2008). Whilst limitations are associated with behavioural models that study antipsychotic-like effects, such as the reversal of psychotomimetic-induced behaviours, they are widely used to screen novel compounds and are posited to model the positive symptoms associated with schizophrenia, such as hyperdopaminergic or hypoglutamatergic states (Jones et al., 2011). Furthermore these models have previously provided data on the role of mGluR2 vs mGluR3 that have pushed forward our understanding of these receptor systems (Moghaddam and Adams, 1998; Cartmell et al., 2000; Jones et al., 2011). However, despite evidence of efficacy in rodent models, clinical trials analyzing the antipsychotic effects of mGluR2/3 compounds have so far shown limited success (Patil et al., 2007; Kinon et al., 2011).

Group II mGlu receptors (mGluR2/3) have been linked to the regulation of sleep and sleep disturbances, and changes in sleep-wake architecture are observed in psychiatric disorders (Benca et al., 1992; Breslau et al., 1996; Krystal et al., 2008). Glutamatergic neurotransmission is suggested to be critical in regulating the arousal system (Feinberg and Campbell, 2008), with cortical glutamate levels tightly regulated between wake and different sleep states (Lopez-Rodriguez et al., 2007) and mGluR2/3 activity has been shown to regulate these levels (Lorrain et al., 2003). Pharmacological studies have shown reduced REM sleep following mGluR2/3 agonist treatment (Feinberg et al., 2002), an effect suggested to be mGluR2 dependent (Ahnaou et al., 2009). Both an mGluR2/3 antagonist and mGluR2 negative allosteric modulator (NAM) increased arousal and wakefulness (Feinberg et al., 2005; Ahnaou et al., 2014), further supporting the role of mGluR2/3 in sleep-wake architecture. Recent work has also shown that knockout of both mGluR2 and mGluR3 in mice results in fragmented sleep and circadian changes (Pritchett et al., 2015).

The interplay of both excitatory and inhibitory neurotransmission and their fluctuating activity is critical in the generation of network oscillations and are considered critical for normal CNS function (Tamas et al., 2000; Buzsaki and Draguhn, 2004; Sohal et al., 2009), with early work highlighting the importance of mGluRs in this control (Whittington et al., 1995). Pharmacological studies have suggested the involvement of mGluR2/3 in oscillatory activity, with suppression of theta and gamma oscillations by mGluR2/3 agonism (Feinberg et al., 2002; Jones et al., 2012) and a marked activation of the same oscillations following mGluR2/3 antagonism (Feinberg et al., 2005; Ahnaou et al., 2014). Recent work has indicated that the mGluR2/3 agonist LY379268 and an mGluR2 positive allosteric modulator (PAM) TASP0443294 can attenuate NMDA antagonist induced aberrant gamma oscillations (Hiyoshi et al., 2014; Hikichi et al., 2015), suggesting the importance of mGluR2 in the effects. Nonetheless, far less is known of the subtype selective role of mGluR2 or mGluR3 in other oscillation bands, with these current data investigating the role of mGluR2 and mGluR3 across multiple oscillation bands.

Despite their obvious potential as novel drug targets, progress to the clinic with group II ligands has not yet been successful with recent failures of the mixed mGluR2/3 agonist LY2140023 in Phase II and Phase III clinical trials for schizophrenia (Kinon et al., 2011; Downing et al., 2014; Maksymetz et al., 2017). One of the challenges is that orthosteric compounds currently available act at both mGluR2 and mGluR3 subtypes. Studies in knockout mice have been useful in discerning the role of mGluR2 and mGluR3, although some mixed results have been observed, possibly linked to their genetic background and compensatory expression (Higgins et al., 2004; Lyon et al. 2008, 2011; Lainiola et al., 2014; De Filippis et al., 2015). We recently reported on a novel Han Wistar rat strain containing a nonsense mutation in the *Grm2* gene that leads to a loss of mGluR2 expression (Wood et al., 2017). In this study, we have used these Han Wistar rats to investigate the role of mGluR2 and mGluR3 receptors in psychostimulant-induced hyperlocomotion, sleep-wake architecture and cortical network oscillations as measured by EEG. By further characterizing the role of these receptors using the Han Wistar rats we aim to provide more information on the subtype specific role of mGluR2 and mGluR3.

## 2 Methods

### 2.1 Subjects

Male adult HSD Han Wistar rats (HSD:Wi, Harlan UK) and Wistar (Crl:WI, Charles River, UK) were used for all experiments. Both strains are outbred lines of Wistar origin but have been bred separately for approximately 80 years (Wood et al., 2017). All studies were conducted in accordance with the Animals (Scientific Procedures) Act 1986 and University of Bristol and Eli Lilly UK ethical review. Food (Laboratory chow, Purina, UK) and water were available *ad libitum* for all studies.

### 2.2 Locomotor activity and rearing analysis

All rats were habituated to a wooden arena (90 cm x 100 cm) with 50 cm high black walls and flooring for five 30 min sessions before the study. On test days, after 10 min in the arena the rats were pretreated with an intraperitoneal (i.p) injection of LY354740 10 mg/kg or distilled water and then after 30 min were administered amphetamine (3 mg/kg), phencyclidine (PCP; 6 mg/kg) or 0.9% sterile saline i.p. followed by an hour of behavioural analysis. The arena was cleaned between rats using 70% ethanol.

Locomotor activity was tracked by a video camera with Ethovision Software (Noldus, US) whilst rearing behaviours were scored online by an individual blind to strain. Total distance moved (cm) and total rearing behaviour during the testing period were calculated. This experiment used a within-subject Latin-square design with treatments and strain counterbalanced across testing days, with one week washout between testing sessions. Doses of amphetamine, PCP and LY354740 were selected based on previously published studies (Castellani and Adams, 1981; Antoniou et al., 1998; Cartmell et al., 1999).

### 2.3 EEG sleep/wake measures

#### 2.3.1 Surgery

Chronic measurement of EEG and electromyogram (EMG) was conducted using cranial implants placed under anaesthesia previously described by Seidel et al. (1995). The implant consisted of a miniature connector (Omnetics, USA) connected to five stainless steel screws positioned from bregma, with two frontal (+3.5 mm AP, ± 2 mm ML) and two occipital screws (−6.5 mm AP, ± 5.2 mm ML) for EEG recording and one overlying the cerebellum to be used as a ground. The implant housed two Teflon-coated stainless steel wires placed under each nuchal trapezoid muscle for EMG recordings. A miniature transmitter (Minimitter PDT4000G, Philips Respironics, Bend, OR) to monitor body temperature and locomotor activity (LMA) was placed in the abdomen during the same surgery. Analgesics were used to minimize pain, buprenorphine (0.05 mg/kg, SC) was administered pre-operatively, at the end of the surgery day and on the morning of the first post-operative day, and Metacam (meloxicam, 0.15 mg/kg, PO) was administered for 6 days after surgery. An antibiotic (Ceporex (cefalexin) 20 mg/kg PO) was administered 24 h before and again immediately before surgery, and for 7 days after surgery. Rats were allowed to recover for at least 28 d prior to experimentation.

#### 2.3.2 Apparatus and drug study protocol

Rats were individually housed within a specially modified polycarbonate cage with a flexible tether connecting the cranial implant to a commutator (Hypnion, USA). Each cage had individually controlled temperature and humidity with a strictly controlled 24 h light-dark cycle (12 h:12 h, 5am lights on). Food and water were available *ad libitum* and consumption of each was measured via the break of infrared beams positioned in front of the food hopper and lixit. Drug treatments occurred 5 h after lights on (Zeitgeber time 5; ZT5), and rats were left undisturbed for 48 h before and after each treatment with at least 6 days between treatments. Drug studies were conducted using a fully crossed over split-plot design whereby each animal was randomly assigned to a treatment sequence.

#### 2.3.3 Data analysis

EEG signals, recorded as the differential between a contralateral pair (right hemisphere frontal and left hemisphere occipital) of skull screws, were amplified 10,000×, bandpass filtered at 1–300 Hz and digitized at 400 Hz [Grass Corp., Quincy, MA]. EMG signals were amplified 20,000×, bandpass filtered at 10–100 Hz and integrated based on the root mean square (RMS). EEG and EMG data were used in combination for on-line classification of arousal states into NREM sleep, REM sleep, or wake in 10s epochs. Wakefulness and sleep states were determined using SCORE-2000™ (Van Gelder et al., 1991), with 10 s epoch of EEG/EMG data classified as wake, NREM or REM sleep. Baseline wake, NREM sleep and REM sleep were calculated for 24 h prior to dosing with post-treatment values calculated for 19 h post treatment. Wake epochs were analysed across multiple band frequencies including delta (0.1–4 Hz), theta (4–9 Hz), beta (12–30 Hz), gamma (30–80 Hz) and high frequency oscillation (130–160 Hz) band power during the main treatment induced wake period. Quality control of the arousal state scoring was facilitated by visual assessment of the raw EEG and EMG signals by experts who were not involved in the data acquisition phase and were blinded with respect to treatment group.

### 2.4 Drugs

Phencyclidine hydrochloride (Tocris, UK) and d-amphetamine sulphate (Sigma, UK) were dissolved in sterile 0.9% saline. To test the effects of the mutation on responses to prototypical mGluR2/3 agonists, we tested LY354740 and LY379268 in locomotor tests and sleep/wake studies respectively. Both compounds are mixed mGluR2/3 agonists and have previously been shown to have similar effects in behavioural models and sleep studies (Moghaddam and Adams, 1998; Cartmell et al., 1999; Woolley et al., 2008; Feinberg et al., 2002; Ahnaou et al., 2009) and are used as reference compounds in the two different establishments where the experiments were undertaken. LY354740 (10 mg/kg i.p), LY3020371 (3 & 10 mg/kg i.p.) and LY379268 (3 & 10 mg/kg p.o.) were provided by Eli Lilly UK (Erl Wood, UK) and dissolved in sterile distilled water with pH corrected to pH7.4 using small additions of 1 M NaOH or 1 M HCl. All drugs were made up fresh each testing day and administered at a final volume of 1 ml/kg.

### 2.5 Statistical analysis

Presence and absence of the cys407* mutation in the Han Wistar and Wistar rats used in these experiments was determined using previous methods (Wood et al., 2017).

Locomotor and rearing data were analysed using a mixed three-way repeated measures ANOVA with pretreatment (LY354740 vs vehicle) and treatment (Amphetamine vs vehicle, PCP vs vehicle) as within subject factors and strain as the between subject factor. *Post hoc* independent samples t-tests for between strain analyses or pairwise comparisons of treatment effects were reported.

A fast Fourier transform of the EEG signal produced a measure of the spectral power for each discreetly scored 10-s epoch and subsequently binned into the frequency bands: delta (0.1–4 Hz), theta (5–9 Hz), beta (12–30 Hz), gamma (30–80 Hz) and high frequency oscillations (130–160 Hz). For spectral data, the effects of the two compounds were evaluated over the first 7-h light phase following treatment when the wake promoting effects were maximal, with total accumulated power analysed over that period. Statistical analyses of sleep and spectral parameters were performed using the SAS (version 9.2, SAS Institute, Inc., Cary, NC) software package.

Analysis of the sleep dependent variables was conducted over the post treatment period light phase (ZT5-ZT12) and dark phase (ZT13- ZT24), with a mixed model analysis of covariance with treatment and strain fixed effects, with contrasts used to test for statistical significance. The change from vehicle for each dose within each strain, and the treatment*strain interaction were evaluated, with each subject acting as its own control (split-plot crossover study design) and a Tukey's multiple comparison adjustment applied. Similarly, the estimated difference in the effect of LY treatments between strains were also analysed and adjusted. Least squares mean differences and standard errors are reported.

For the accumulated spectral power data, the mixed model procedure was used to perform an analysis of covariance with treatment, strain and treatment*strain fixed effects. Comparisons of individual treatments for each strain or between strains were completed as appropriate with each subject acting as its own control and a Tukey's multiple comparison adjustment. Each subject acted as its own control and a Tukey's multiple comparisons adjustment applied. Least squares mean differences and standard errors are reported.

Baseline characteristics (chambers, body weight, age, pre-treatment distributions) were checked for homogeneity across treatments, and factors of Body Weight, Age, Pre*Dose interaction, as well as 2- and 3-way RunDate interactions were used as covariates within the model for influence on final estimates.

All data are displayed as means ± SEM, with all figures created using GraphPad Prism 6 (GraphPad Software, US). All tests of significance were performed with α = 0.05.

## 3 Results

### 3.1 Effects of mGluR2/3 agonist LY354740 on amphetamine and phencyclidine-induced locomotion and rearing behaviours

**Fig. 1:**
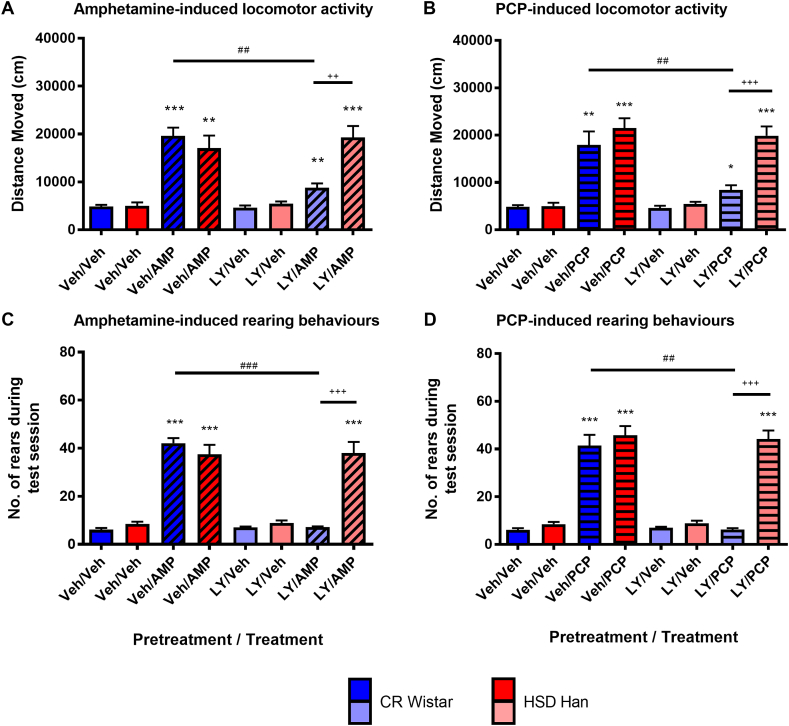
**Effects of mGluR2/3 agonist LY354740 (10 mg/kg) on amphetamine (3 mg/kg) and phencyclidine (6 mg/kg) induced behaviours in Wistar and Han Wistar rats.** Amphetamine (AMP) and phencyclidine (PCP) increased locomotor activity (A, B) and rearing behaviours (C, D) in both Wistar (blue/light blue) and Han Wistar (red/pink) rats. Pretreatment with LY35740 (LY) significantly attenuated the behavioural responses to amphetamine and PCP in the Wistar rats (light blue) compared to vehicle pretreatment (Veh; blue), which did not occur in the Han Wistar rats (pink, LY pretreatment; red, Veh pretreatment). Individual data bars indicate mean values ± SEM for specific pretreatment/treatment combinations (e.g. LY/AMP – LY354740 pretreatment followed by amphetamine treatment), with strain and pretreatment combinations separated by colour as described. Significant post hoc comparisons are highlighted, with comparison to vehicle (*), between strains (+) or pretreatment (#) displayed, with significance values of p < 0.05*, p < 0.01** and p < 0.001***. (For interpretation of the references to colour in this figure legend, the reader is referred to the Web version of this article.)

Administration of the psychostimulants amphetamine (AMP; 3 mg/kg) and PCP (6 mg/kg) increased locomotor activity (Fig. 1A and B) and rearing behaviours (Fig. 1C and D) in both Han Wistar (red) and Wistar rats (blue) with a main effect of treatment observed (AMP, locomotor activity F_(1,14)_ = 76.5, p < 0.001, rearing F_(1,14)_ = 109.6, p < 0.001; PCP, locomotor activity F_(1,14)_ = 78.4, p < 0.001; rearing F_(1,14)_ = 196.0, p < 0.001). Post-hoc pairwise comparisons revealed an increase in both behaviours for both strains and compounds (p < 0.01 for all comparisons).

LY354740 (10 mg/kg) pretreatment attenuated amphetamine- and PCP-induced locomotor activity (Fig. 1A and B) in the Wistar rats (light blue) but not Han Wistar rats (pink). Significant strain and pretreatment effects were observed for the PCP locomotor data (strain, F_(1,14)_ = 6.801, p = 0.021; pretreatment, F_(1,14)_ = 25.3, p < 0.001) in addition to multiple interactions (pretreatment*strain, F_(1,14)_ = 15.5, p = 0.001; pretreatment*treatment*strain, F_(1,14)_ = 7.76, p = 0.015). A trend towards a significant strain effect was observed for amphetamine (F_(1,14)_ = 3.65, p = 0.077), whilst a significant pretreatment effect and multiple interactions were observed (pretreatment, F_(1,14)_ = 6.11, p = 0.027; pretreatment*strain, F_(1,14)_ = 16.5, p = 0.001; pretreatment*treatment*strain, F_(1,14)_ = 15.5, p = 0.001).

LY354740 attenuated both amphetamine and PCP-induced rearing in the Wistar rats only (Fig. 1C and D). Significant main strain and pretreatment effects were observed for both compounds (AMP, strain, F_(1,14)_ = 13.6, p = 0.027; pretreatment, F_(1,14)_ = 88.4, p < 0.001; PCP, strain, F_(1,14)_ = 30.7, p < 0.001; pretreatment, F_(1,14)_ = 34.2, p < 0.001), in addition to significant interactions for both amphetamine and PCP (AMP, pretreatment*strain F_(1,14)_ = 98.2, p < 0.001, pretreatment*treatment*strain, F_(1,14)_ = 88.3, p < 0.001; PCP, pretreatment*strain F_(1,14)_ = 30.0, p < 0.001; pretreatment*treatment*strain F_(1,14)_ = 34.7, p < 0.001).

Post-hoc comparisons for both behaviours indicated the reversal of amphetamine and PCP induced behaviours in the Wistar rats only (AMP, locomotion, p = 0.002; rears, p < 0.001; PCP, locomotion, p = 0.002; rears, p = 0.001), in addition to the strain difference in LY354740 effects (AMP, locomotion p = 0.001; all other analyses p < 0.001). No strain*treatment effects were observed for either compound or behaviour, as similar levels of behaviour were induced for both strains. Furthermore, LY354740 pretreatment alone did not significantly influence either behaviour in either strain (p > 0.05).

### 3.2 Effects of mGluR2/3 agonist LY379268 (3, 10 mg/kg) on sleep-wake architecture and NREM delta activity

**Fig. 2:**
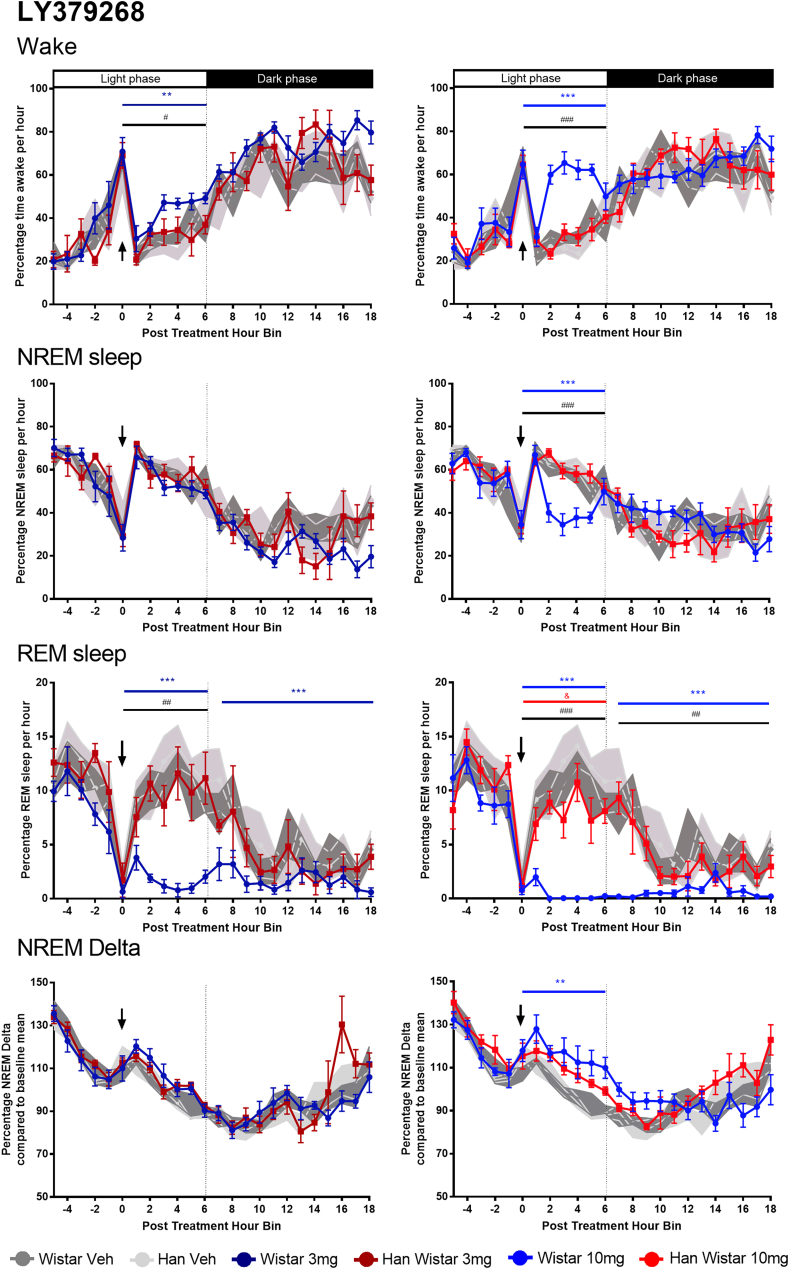
**Effects of mGluR2/3 agonist LY379268 (3, 10 mg/kg) on sleep variables and NREM delta in Wistar and Han Wistar rats.** The effects of LY379268 are split across four variables, wakefulness, NREM sleep, REM sleep and NREM delta activity, with individual time courses for each and different colour lines for each treatment/strain combination. All animals were dosed at ZT5 (post treatment hour 0) as indicated by the arrow, with light and dark phases indicated at the top of the wake graphs. Each data point represents mean ± SEM (Wistar, n = 6–7; Han Wistar, n = 5–6) with significant treatment effects for Wistar rats (*) and strain differences (#) indicated for the relevant time period. All significance values used are p < 0.05*, p < 0.01** and p < 0.001***. (For interpretation of the references to colour in this figure legend, the reader is referred to the Web version of this article.)

The mGluR2/3 agonist LY379268 had divergent effects on sleep in the Wistar and Han Wistar rats (Fig. 2). The most prominent effect was the dose dependent loss of REM sleep in Wistar rats across both the initial light phase (3 mg/kg, F_(1,15.7)_ = 53.1, p < 0.001; 10 mg/kg, F_(1,16.1)_ = 87.1, p < 0.001) and the subsequent dark phase (3 mg/kg, F_(1,15.4)_ = 21.9, p = 0.003; 10 mg/kg, F_(1,15.7)_ = 58.9, p < 0.001). REM sleep loss was greatly reduced in the Han Wistar rats, with significant REM inhibition only present following the 10 mg/kg dose during the initial light period (F_(1,16.3)_ = 14.6, p = 0.015). These differences in response to LY379268 were shown by significant strain effects and strain*treatment interactions at 3 mg/kg and 10 mg/kg during the light phase (strain, 3 mg/kg, F_(1,26.8)_ = 25.9, p = 0.001, 10 mg/kg, F_(1,25.2)_ = 28.6, p < 0.001; strain*treatment F_(1,16.6)_ = 10.0, p = 0.006, F_(1,17)_ = 10.1, p = 0.006; respectively) and 10 mg/kg during the dark phase (strain, F_(1,21.8)_ = 16.8, p = 0.009; strain*treatment, F_(1,16.2)_ = 10.8, p = 0.005).

LY379268 dose dependently increased wakefulness during the light period in the Wistar rats only (3 mg/kg, F_(1,19)_ = 15.5, p = 0.008; 10 mg/kg, F_(1,19)_ = 58.2, p < 0.001). In contrast no effects were seen in the Han Wistar rats, resulting in significant strain effects and strain*treatment interactions during the light phase for both doses (strain, 3 mg/kg, F_(1,28)_ = 11.5, p = 0.028, 10 mg/kg, F_(1,28)_ = 52.6, p < 0.001; strain*treatment 3 mg/kg, F_(1,21.3)_ = 4.6, p = 0.043; 10 mg/kg, F_(21.1)_ = 20.8, p < 0.001). The increased wakefulness in the Wistar rats returned to control levels after 6 h. These changed occurred with an increase in body temperature in the Wistar rats at 10 mg/kg during the light phase (Supplementary Fig. 1; F_(1,16.6)_ = 24.1, p = 0.002), whilst both doses observed an increase during the dark phase (3 mg/kg, F_(1,16.1)_ = 14.9, p = 0.015; 10 mg/kg F_(1,15.9)_ = 13.7, p = 0.02). Minimal effects on body temperature were seen in the Han Wistar rats. Furthermore, the increased wakefulness did not significantly alter locomotor activity (Supplementary Fig. 1).

In the control Wistar rats alone, 10 mg/kg LY379268 reduced NREM sleep during the initial light phase (F_(1,20.9)_ = 35.3, p < 0.001), whilst no effect was observed in the Han Wistar rats. These differences were supported by a strain effect and strain*treatment interaction at 10 mg/kg (F_(1,28)_ = 39.6, p < 0.001; F_(1,22.7)_ = 17.8, p < 0.001). No effects on NREM sleep were observed at 3 mg/kg for either strain. These effects on wakefulness, NREM and REM sleep occurred without changes to sleep bout number or sleep bout duration (Supplementary Fig. 2).

A small increase in NREM delta was observed in the Wistar rats during the light phase following 10 mg/kg LY379268 (F_(1,11.9)_ = 22.7, p = 0.005), whilst no significant effect was observed in the Han Wistar rats. Nonetheless no significant strain effect or strain*treatment interaction was observed. No effects on NREM delta power were observed for either rat strain following 3 mg/kg LY379268 treatment.

### 3.3 Effects of mGluR2/3 antagonist LY3020371 (3, 10 mg/kg) on sleep-wake architecture and NREM delta activity

**Fig. 3:**
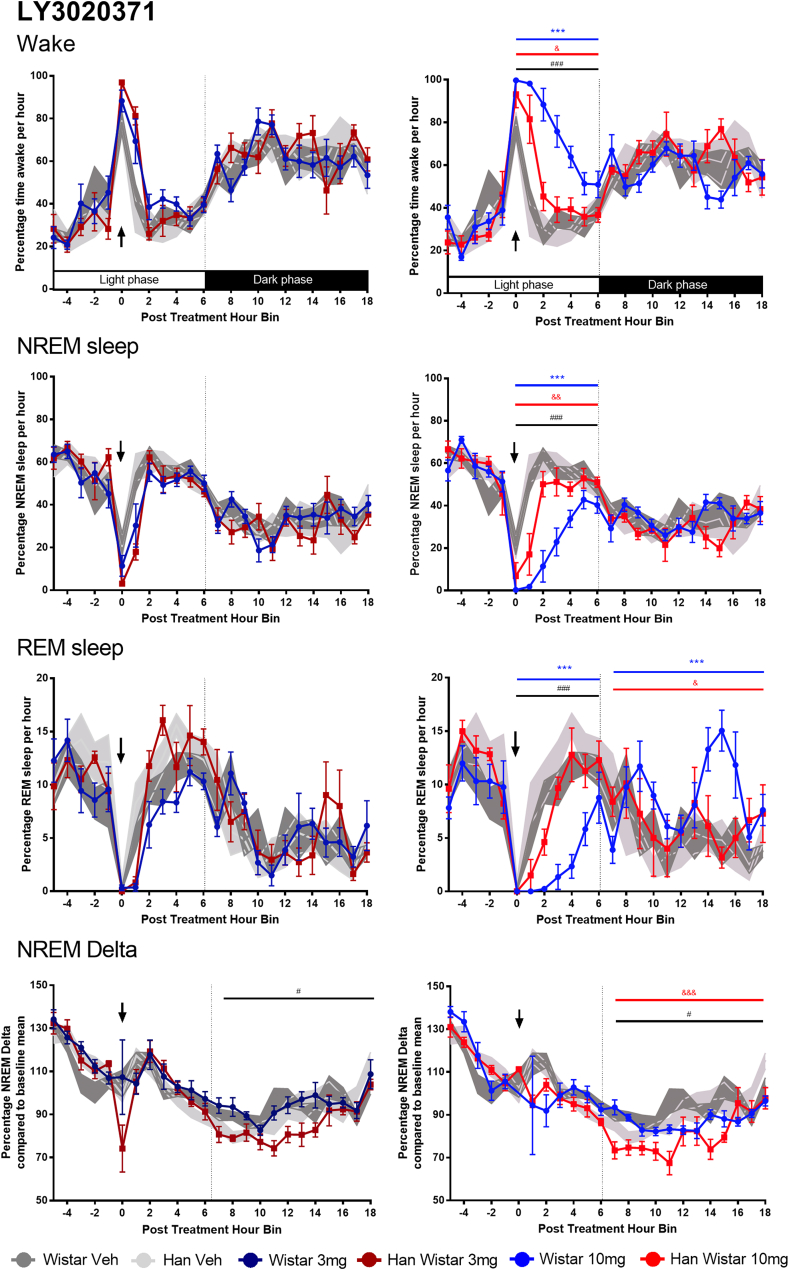
**Effects of mGluR2/3 antagonist LY3020371 (3, 10 mg/kg) on sleep variables and NREM delta in Wistar and Han Wistar rats.** Four variables are reported for the effects of LY3020371, wakefulness, NREM sleep, REM sleep and NREM delta activity. Data are displayed with individual time courses for each variable with different colour lines for each treatment/strain combination. All animals were dosed at ZT5 (post treatment hour 0) as indicated by the arrow, with light and dark phases indicated at the bottom of the wake graphs. Each data point represents mean ± SEM, with significant treatment effects for Wistar rats (*), Han Wistar rats (&) and strain differences (#) indicated for the relevant time period. All significance values used are p < 0.05*, p < 0.01** and p < 0.001***. (For interpretation of the references to colour in this figure legend, the reader is referred to the Web version of this article.)

The mGluR2/3 antagonist LY3020371 had clear wake promoting effects in the Wistar rats at 10 mg/kg (Fig. 3; F_(1,21)_ = 91, p < 0.001), with almost complete sleep loss for 2 h. This treatment effect was also present in the Han Wistar rats during the light phase (F_(1,22.0)_ = 13.1, p = 0.016) but was smaller and over a shorter duration, with a significant strain effect and strain*treatment interaction at 10 mg/kg (F_(1,29.4)_ = 29.5, p < 0.001, F_(1,21.9)_ = 13.6, p = 0.001 respectively). No rebound effects on wakefulness were observed during the following dark phase in either strain. Furthermore 3 mg/kg had no effect on any sleep parameter throughout both light and dark phases.

This wake-promotion resulted in a large reduction in NREM sleep in the Wistar rats during the light phase following 10 mg/kg LY3020371 (F_(1,21.5)_ = 102.8, p < 0.001). A smaller reduction in NREM sleep was observed in the Han Wistar rats (F_(1,21.6)_ = 15, p = 0.009), with these strain differences highlighted by both significant strain and strain*treatment effects (F_(1,30.0)_ = 24.1, p < 0.001, F_(1,21.9)_ = 15.2, p < 0.001 respectively). Minimal effects were seen with 3 mg/kg LY379268 and despite the loss of NREM sleep by 10 mg/kg, little rebound NREM sleep was observed during the following dark phase in either strain. This wake promotion during the light phase by 10 mg/kg LY3020371 resulted in increased locomotor activity and resultant body temperature in the Wistar rats alone (Supplementary Fig. 3; locomotor activity, F_(1,17.6)_ = 38.2, p < 0.001; body temperature, F_(1,16.6)_ = 28.5, p < 0.001), with minimal effects in the Han Wistar rats. Furthermore the increased wakefulness resulted in reduced sleep bout number and bout duration during the light phase for the Wistar rats alone (Supplementary Fig. 4; bout number, F_(1,19.4)_ = 14.5, p = 0.012; bout duration, F_(1,22.4)_ = 6.5, p = 0.018).

**Fig. 4:**
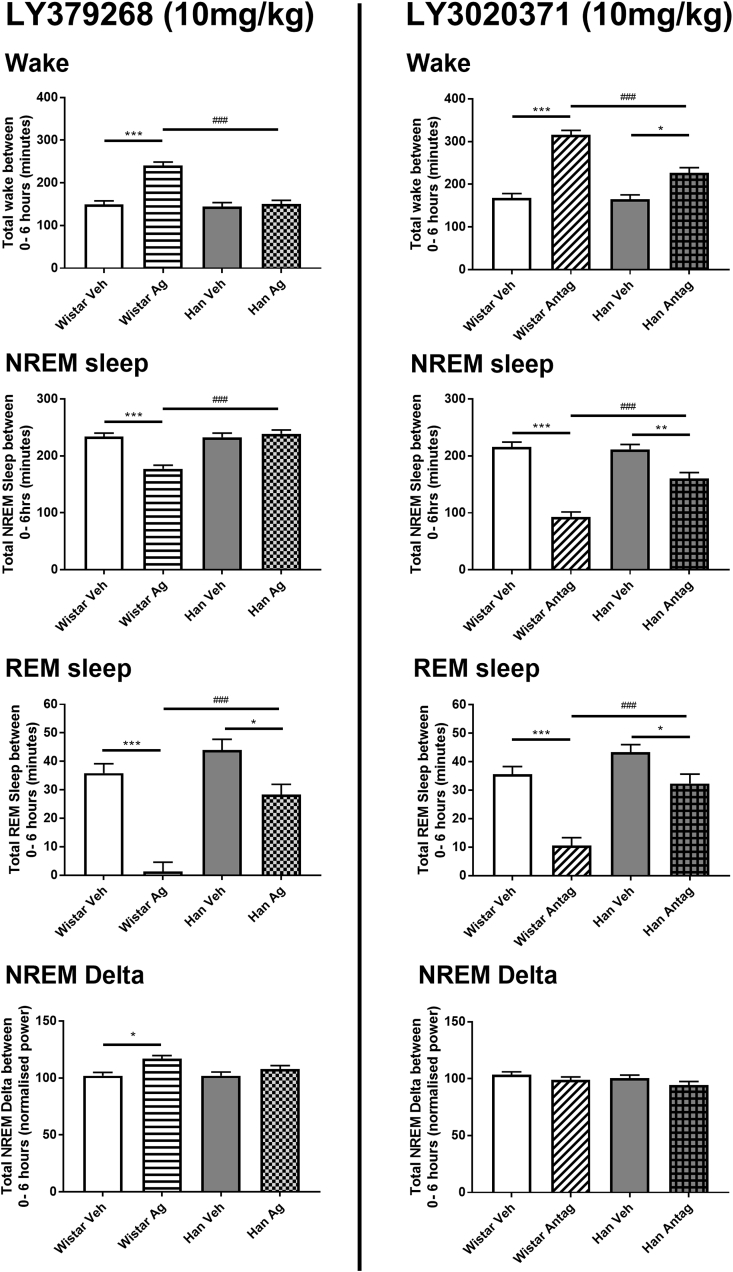
**Histogram summary of mGluR2/3 receptor agonist LY379268 (10 mg/kg) and antagonist LY3020371 (10 mg/kg) effects on accumulated sleep variables and NREM delta in Wistar and Han Wistar rats.** Four variables that are reported for both compounds, wakefulness, NREM sleep, REM sleep and NREM delta activity. These data are the accumulated amount of sleep or delta activity during the 7 h post treatment light period (0–6 h in Fig. 2, Fig. 3) and are displayed as mean ± SEM. Significant treatment effects (*) and strain differences (#) are indicated as appropriate using significance values of p < 0.05*, p < 0.01** and p < 0.001***.

During the light phase, significant reductions in REM sleep were only observed in the Wistar rats following 10 mg/kg LY3020371 (F_(1,19.5)_ = 39.2, p < 0.001). Han Wistar rats showed a small but non-significant reduction throughout the light period, although total accumulated REM sleep over the 6 h post treatment was significantly reduced relative to the previous 24 h baseline (Fig. 4; p = 0.005). Nonetheless, these strain differences in response throughout the light period were indicated by significant a strain effect and strain*treatment interaction (F_(1,29.6)_ = 25.1, p < 0.001, F_(1,20.3)_ = 5.4, p = 0.031, respectively). Significant rebound REM sleep was observed in both strains, with a far larger rebound in the Wistar rats during the following dark phase compared to the Han Wistar rats, as evidenced by significant strain*treatment interaction (F_(1,19.5)_ = 4.4, p = 0.049) as well as treatment effects for both Wistar (F_(1,19.8)_ = 46.1, p < 0.001) and Han Wistar strains (F_(1,18.7)_ = 11.5, p = 0.03), however no strain effect was observed.

NREM delta activity was initially reduced in both rat strains, however delta activity returned to vehicle levels 2 h post treatment. No significant treatment effects were observed for the initial post treatment light phase (Fig. 3) as well as no difference in accumulated delta power (Fig. 4). During the subsequent dark phase, Han Wistar rats had reduced NREM delta activity following 10 mg/kg LY3020371 treatment (F_(1,19.7)_ = 25.1, p < 0.001), with no effect at 3 mg/kg in the Han Wistar rats or at either dose in the Wistar rats. A significant strain effect and strain*treatment interaction was observed for NREM delta during the dark phase (F_(1,27.6)_ = 13.2, p = 0.018; F_(1,19.7)_ = 5.1, p = 0.035).

### 3.4 Analysis of wake EEG spectra following mGluR2/3 agonist LY379268 (3, 10 mg/kg) treatment

**Fig. 5:**
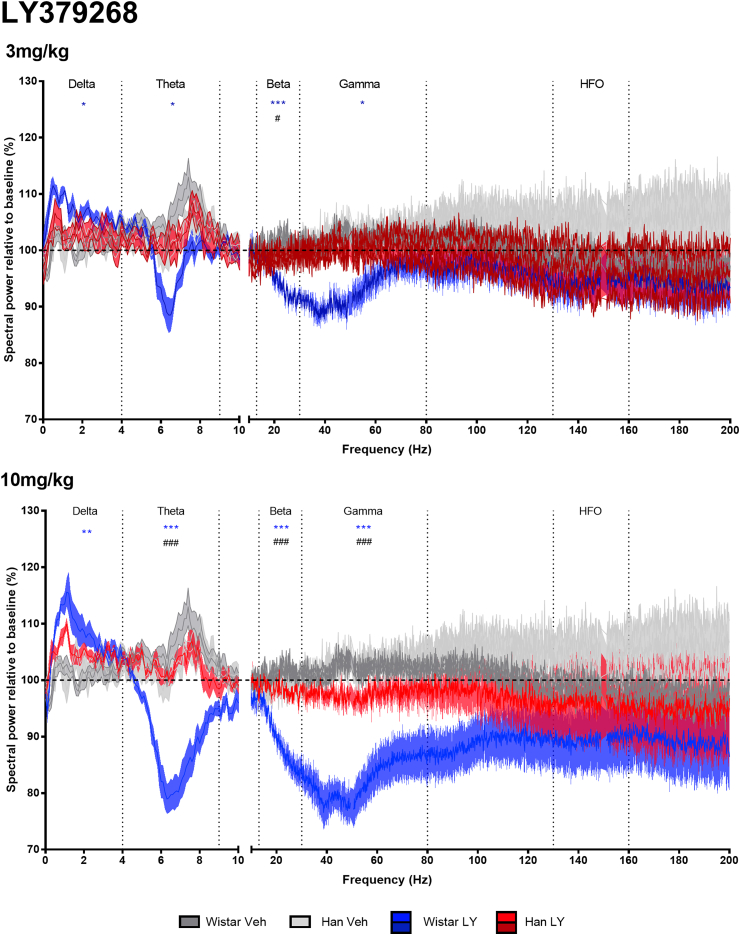
**Wake spectral power analysis of mGluR2/3 agonist LY379268 (3, 10 mg/kg) or vehicle administration at ZT5 in Wistar and Han Wistar rats.** Spectral power data was normalized relative to a 24 h baseline period, with data as represented as means for individual Hz with ± shaded SEM values, with separate lines for Wistar and Han Wistar rats following vehicle (dark grey/grey) or LY379268 (blue/red) treatment. Oscillation bands are labeled and separated by vertical dotted lines. Strain specific drug effects are indicated for the Wistar (*) and Han Wistar rats (&), whilst significant strain differences in drug response are also indicated (#) using significance values of p < 0.05*, p < 0.01** and p < 0.001***. (For interpretation of the references to colour in this figure legend, the reader is referred to the Web version of this article.)

Treatment with LY379268 altered the wake EEG spectra of the Wistar rats across multiple power bands during the post treatment wakefulness period (first 7 h after treatment), with more limited effects in the Han Wistar rats (Fig. 5).

LY379268 increased delta power (0.1–4 Hz) in the Wistar with minimal effects in the Han Wistar rats, with a peak between 0.5 and 1 Hz for both doses. This increase in delta power showed a main overall effect of treatment (F_(2,30)_ = 8.36, p = 0.0013), but not strain or treatment*strain effects (F_(1,30)_ = 3.86, p = 0.0587, F_(2,30)_ = 2.39, p = 0.1090 respectively). In individual doses, delta power was increased at both 3 mg/kg and 10 mg/kg in the Wistar rats (p = 0.0135 p = 0.0021 respectively) following adjustments for multiple comparisons, whilst neither dose altered delta in the Han Wistar rats.

Theta power (4.1–9 Hz) in the Wistar rats was reduced by LY379268 with minimal effect in the Han Wistar rats. These effects were supported by main treatment, strain and treatment*strain effects for theta power (F_(2,30)_ = 17.75, p < 0.001, F_(1,30)_ = 16.49, p < 0.001; F_(2,30)_ = 16.96, p < 0.001 respectively), with reduced power in the Wistar rats at both doses (3 mg/kg, p = 0.0010; 10 mg/kg, p < 0.001), which was not observed in the Han Wistar rats. Strain comparisons indicated a difference at the 10 mg/kg dose only for theta power (p < 0.001).

A dose dependent reduction in beta power (12–30 Hz) was observed in only the Wistar rats, with significant main effects and interactions observed (treatment, F_(2,30)_ = 30.16, p < 0.001; strain, F_(1,30)_ = 26.64, p < 0.001; treatment*strain, F_(2,30)_ = 22.87, p < 0.001). The response to LY379268 was different between strains at 3 mg/kg and 10 mg/kg (p = 0.0468, p < 0.001, respectively), with Wistar rats reducing beta power at both doses (p < 0.001 for both analyses), whilst neither dose of LY379268 altered beta power in the Han Wistar rats.

In the higher frequency gamma band (30.1–80 Hz), LY379268 reduced spectral power in the Wistar rats but not Han Wistar rats, with significant main effects and interactions (treatment, F_(2,30)_ = 18.98, p < 0.001; strain, F_(1,30)_ = 20.15, p < 0.001; treatment*strain, F_(2,30)_ = 6.61, p = 0.0042). Further analysis indicated these effects resulted from both doses reducing gamma power (3 mg/kg, p = 0.0466 and 10 mg/kg, p < 0.001) with a strain difference observed at 10 mg/kg (p < 0.001). LY379268 had no effect on the higher frequency HFO (130–160 Hz) band in either of the strains.

### 3.5 Analysis of wake EEG spectra following mGluR2/3 antagonist LY3020371 (3, 10 mg/kg) treatment

**Fig. 6:**
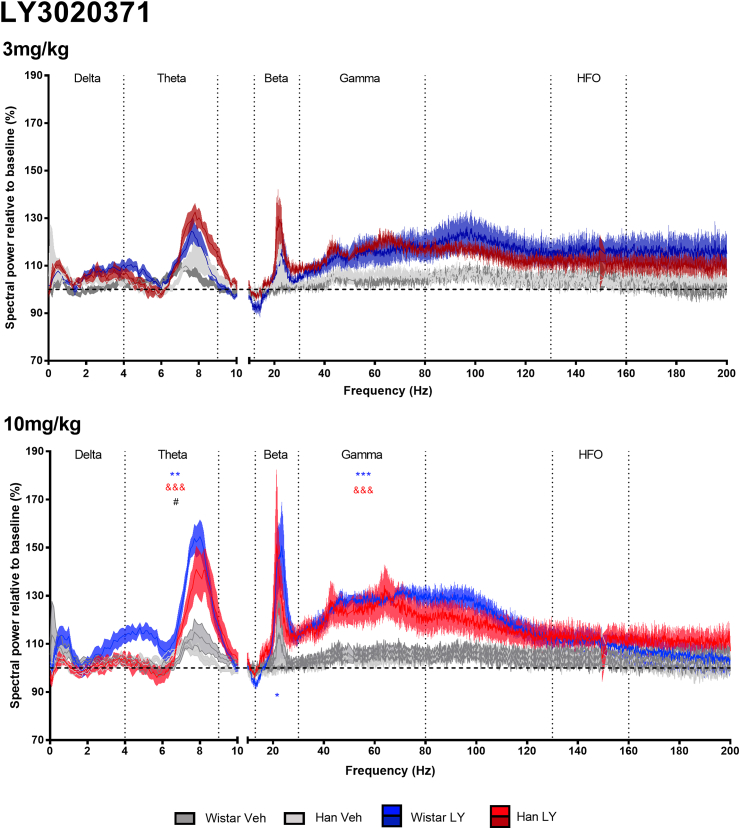
**Wake spectral power analysis of mGluR2/3 antagonist LY3020371 (3, 10 mg/kg) or vehicle administration at ZT5 in Wistar and Han Wistar rats.** Spectral power data was normalized relative to a 24 h baseline period, with data as represented as means for individual Hz with ± shaded SEM values, with separate lines for Wistar and Han Wistar rats following vehicle (dark grey/grey) or LY3020371 (blue/red) treatment. Oscillation bands are labeled and separated by vertical dotted lines. Strain specific drug effects are indicated for the Wistar (*) and Han Wistar rats (&), whilst significant strain differences in drug response are also indicated (#) using significance values of p < 0.05*, p < 0.01** and p < 0.001***. (For interpretation of the references to colour in this figure legend, the reader is referred to the Web version of this article.)

LY3020371 increased theta power in both the Wistar and Han Wistar rats at the 10 mg/kg dose but not 3 mg/kg (Fig. 6). These theta effects saw both main treatment and treatment*strain effects, however no main strain effects were observed (treatment, F_(2,32)_ = 25.86, p < 0.001; treatment*strain, F_(2,32)_ = 5.40, p = 0.0096; strain, F_(2,32)_ = 3.46, p = 0.072). Further comparisons indicated LY3020371 increased theta power at 10 mg/kg in both the Wistar rats (p < 0.001) and Han Wistar rats (p < 0.001), with a significantly larger increase in the Wistar rats at this dose with a strain difference observed (p = 0.0102).

Administration of LY3020371 resulted in sharp peaks in beta power with main treatment effects observed but no strain effect or treatment*strain interaction (treatment, F_(2,32)_ = 7.69, p = 0.0019; strain, F_(1,32)_ = 0.84, p = 0.3657; treatment*strain, F_(2,32)_ = 0.47, p = 0.6303). Individual treatment comparison revealed that LY3020371 dosed at 10 mg/kg increased beta power in the Wistar rats only relative to vehicle controls (p = 0.0258).

A small increase in gamma band power was observed in both rat strains, with overall main treatment effects (F_(2,32)_ = 23.23, p < 0.001) but no significant strain effects (F_(2,32)_ = 0.02, p = 0.9001) or treatment*strain interactions (F_(2,32)_ = 0.47, p = 0.6314). Individual dose comparisons showed that gamma power increased in both the Han Wistar (p = 0.0042) and Wistar (p < 0.0001) rats at the 10 mg/kg dose only, and that the increase was equivalent in both strains (p = 0.98).

LY3020371 had no effect on delta power or in the higher frequency HFO (130–160 Hz) band in either of the strains.

## 4 Discussion

These data provide evidence for distinct roles mediated by mGluR2 versus mGluR3 in relation to the actions of mixed mGluR2/3 agonists. The ability of these drugs to attenuate both amphetamine and PCP-induced hyperlocomotion and rearing behaviours was lost in the mutant Han Wistar rat indicating a critical role for the mGluR2 subtype in these behavioural effects. Utilizing these mutant rats, we were also able to provide new insights into the possible roles that mGluR2 and mGluR3 contribute to sleep-wake architecture and EEG spectral data. The mGluR2 subtype was found to be responsible for the REM sleep suppression observed following mGluR2/3 agonist LY379268 treatment. An mGluR2 specific increase in wake delta power but reduced power in all other frequency bands was also observed however, the presence of a reduced but not total loss of effect in the Han Wistar rats suggests a contributory role for the mGluR3 subtype.

The results for the mGluR2/3 antagonist LY3020371 (Witkin et al., 2017) revealed increase in arousal in both rat strains with increased wakefulness, and reduced NREM and REM sleep. Although clearly an mGluR2 and mGluR3 mediated response, the results for the Han Wistar animals showed an attenuation suggesting that the absence of mGluR2 reduces the overall effect of the antagonist. Despite the sleep loss, little rebound in NREM sleep was observed for either strain. LY3020371 increased spectral power in Wistar rats at lower frequencies (>10 Hz), with no significant effects in Han Wistar rats, whilst a specific peak in beta power was observed in the Han Wistar strain only. At higher frequencies (>30 Hz), LY3020371 increased gamma and HFO band power in both rat strains.

### 4.1 mGluR2 plays a key role in the attenuation of psychostimulant-induced hyperlocomotion observed with mixed mGluR2/3 agonists

Despite the lack of mGluR2 expression in the Han Wistar strain, no baseline differences in the response to amphetamine or PCP were observed (Fig. 1). These data suggest mGluR2 is not involved in a modulatory role under normal conditions despite their proposed involvement in processes which may drive these behaviours e.g. amphetamine induced dopamine release within the nucleus accumbens (Millan et al., 1999) or PCP-induced release of glutamate in the prefrontal cortex (PFC; Takahata and Moghaddam, 2003). This is consistent with results in mGluR2^−/−^ mice (Fell et al., 2008; Woolley et al., 2008) that also failed to show any differences in sensitivity to the stimulant effects of either treatment. However, mGluR2^−/−^ mice show baseline locomotor differences in some circumstances although these effects are not consistent and are task- or arousal state-dependent (Morishima et al., 2005; De Filippis et al., 2015). The reasons for these differences are not clear as both animals lack a functional receptor. Species differences may be a factor as compensatory mechanisms in knockout mice may differ from those seen in rats containing a spontaneous mutation, whilst there are also differences in the assays used to assess locomotor activity. It is also important to consider that the reversal of locomotor effects does not appear to be linked to changes in arousal. We have seen an increase in wakefulness following treatment with the mGluR2/3 agonist LY379268 (Fig. 2). When administered alone LY354740 did not significantly alter locomotor activity in either strain (Fig. 1) and LY379268 had no effect on activity recorded during the sleep study (Supplementary Fig. 1). This suggests that whilst mGluR2/3 agonists may alter the arousal of the rats their effects on locomotor activity and rearing may relate more specifically to those induced by psychotomimetics.

The reversal of both hyperlocomotion and rearing behaviours by LY354740 in the Wistar rats agrees with the proposed antipsychotic-like effects of mGluR2/3 agonists (Moghaddam and Adams, 1998; Gewirtz and Marek, 2000; but also see Cartmell et al., 1999). The lack of a reversal in the Han Wistar rats supports mGluR2 as the specific receptor for these effects, which is consistent with previous data using subtype selective modulators and knockout mice (Galici et al., 2005; Woolley et al., 2008).

### 4.2 Specific role for mGluR2 in agonist-induced loss of REM sleep but both mGluR2 and mGluR3 contribute to wake promoting effects following antagonist treatment

Normal sleep-wake architecture was observed in the Wistar and Han Wistar rats following vehicle treatment, as no differences were observed between strains (Fig. 2, Fig. 3). This suggests that loss of mGluR2 does not directly affect normal sleep-wake functioning similar to previous work with mGluR2^−/−^ knockout mice (Ahnaou et al., 2009).

The mixed mGluR2/3 agonist, LY379268 suppressed REM sleep in the Wistar rats, with REM loss extending throughout the post treatment light period at both doses tested (Fig. 2). This effect was REM specific at 3 mg/kg, with little effect on wakefulness or NREM sleep, whilst 10 mg/kg increased wakefulness and reduced NREM sleep. These sleep effects were not observed in the Han Wistar rats indicating that mGluR2 is the specific subtype responsible for mediating this effect. This is consistent with a previous report using both knockout mice and mGluR2 PAM biphenyl-indanone A (BINA; Ahnaou et al., 2009). Cortical glutamate is tightly regulated during sleep states with low levels observed during wakefulness and NREM sleep and high levels during REM (Lopez-Rodriguez et al., 2007), therefore activation of mGluR2 and suppression of cortical glutamate may account for the almost total suppression of REM observed. The influence of other neurotransmitters that are regulated by mGluR2 and modulate sleep and arousal, such as acetylcholine, serotonin and GABA, may also be involved (Cartmell and Schoepp, 2000; Kohlmeier et al., 2013) but further studies would be needed. Interestingly activation of mGluR2/3 did not appear to fragment sleep as sleep bout duration and number of sleep bouts were not affected by either dose of LY379268 (Supplementary Fig. 2), suggesting whilst REM sleep is heavily suppressed the Wistar rats normal physiological transitions between sleep and wake remain relatively intact.

The novel, mixed mGluR2/3 antagonist LY3020371 increased arousal and wakefulness in both rat strains (Fig. 3), with larger effects seen in the Wistar rats than Han Wistar rats. Initial effects showed almost complete loss for 2–3 h post treatment which also resulted in a reduction in sleep bout duration and number (Supplementary Fig. 4) as well as a concomitant increase in locomotor activity and body temperature (Supplementary Fig. 3). These data suggest that blockade of mGluR2 and/or mGluR3 can increase wakefulness but with a maximal effect following blockade of both receptors. This agrees with previous reports as the mGluR2/3 antagonist LY341495 dramatically increased wakefulness (Feinberg et al., 2005; Ahnaou et al., 2014), with a smaller wake promoting effect by negative allosteric modulation of mGluR2 (Ahnaou et al., 2014). This increased arousal may stem from mGluR2/3-mediated effects on histaminergic neurons within the hypothalamus (Okakura et al., 1992) as recent data suggested that mGluR2/3 blockade increases histamine release within this region promoting arousal (Fell et al., 2015), which would also result in the fragmentation of sleep that occurs due to large scale blockade of mGluR2/3 by 10 mg/kg LY3020371 (Supplementary Fig. 4). Furthermore, our data indicate that constitutive mGluR3 activity present in the Han Wistar rats may be maintaining sleep-wake functioning, as mGluR3 blockade alone results in increased arousal (Fig. 3).

The sleep data from both the agonist and antagonist indicate that there is an optimal activity at mGluR2 and mGluR3 that facilitate normal sleep-wake architecture. Disruptions of this process can result in changes to this architecture but with differing effects, as widespread mGluR2 activation, the predominant effector for LY379268 (Fig. 2), drives the loss of REM sleep and at higher doses increases wakefulness, whilst inactivation of natural mGluR2 and mGluR3 tone by LY3020371 (Fig. 3), results in a dramatic rise in arousal and wakefulness resulting in an overall non-specific sleep loss.

As sleep is a homeostatic mechanism, sleep restriction through pharmacological or paradoxical methods results in increased sleep pressure and is followed by a period of hypersomnolence during the following wake/active period. These effects are classically seen with wake promoting agents such as methamphetamine (Edgar and Seidel, 1997), however, the wake promoting effects of LY3020371 were not followed by a period of hypersomnolence in either rat strain, although an increase in REM sleep was observed during the dark phase (Fig. 3). A similar lack of a hypersomnolence period was observed with dosing at two hours into the inactive phase (ZT-2; Ahnaou et al., 2014) suggesting mGluR2 or mGluR2/3 blockade can induce a specific wake-promoting effect, without the NREM rebound.

### 4.3 The role of mGluR2 and mGluR3 in oscillations <30 Hz (delta, theta, beta)

LY379268 increased wake delta power in the Wistar but not Han Wistar rats (Fig. 5, Fig. 6; 0–4 Hz) suggesting that activation of mGluR2 increases cortical delta oscillations. Recent data supports this mGluR2-mediated balance of delta activity, as an mGluR2 PAM increased (Siok et al., 2012) and mGluR2 NAM decreased (Ahnaou et al., 2014) delta power. Increases in delta oscillations may be a secondary response to drug-induced wakefulness, however the lack of a delta effect in the Han Wistar rats, despite increased wakefulness, does not support that hypothesis.

Modulation of mGluR2 and mGluR3 influenced theta oscillations as LY379268 decreased theta (Fig. 5) in an mGluR2 dependent manner and conversely LY3020371 increased theta power (Fig. 6) in both rat strains, suggesting involvement of both mGluR2 and mGluR3 in the control of theta oscillations. Similar effects on theta have also been reported previously with group II pharmacology and support the involvement of mGluR2 (Feinberg et al. 2002, 2005; Siok et al., 2012; Ahnaou et al., 2014). As theta oscillations are suggested to be important in cognitive functioning (Basar et al., 2001; Jones and Wilson, 2005), the effects on theta power in these data may explain the suggested cognitive deficits observed in some studies with mGluR2/3 agonists in normal animals (Higgins et al., 2004; Schlumberger et al., 2009). Furthermore, mGluR2/3 antagonists and mGluR2 NAMs have been shown to improve performance in behavioural tasks (Higgins et al., 2004; Goeldner et al., 2013).

The decrease in beta oscillation (12–30 Hz) induced by LY379268 was specific to mGluR2 activation as no effects were observed in the Han Wistar strain (Fig. 5), however it can clearly be seen that this beta decrease is part of the adjoining gamma response therefore may not be beta specific. A sharp increase in beta oscillations was seen in both rat strains following treatment with LY3020371 however a significant effect was only seen in the Wistar rats at 10 mg/kg. Beta oscillations have been associated with locomotor activity (Engel and Fries, 2010; Jenkinson and Brown, 2011), however the peaks in beta oscillations observed were associated with minimal locomotor effect in the Han Wistar rats compared to a dramatic increase in the Wistar rats (Supplementary Fig. 1).

### 4.4 Constitutive activity of mGluR2/3 in gamma and HFO bands with mGluR2 specific suppression by LY379268

Aberrant theta and gamma oscillations have been observed in psychosis-like states (Lisman and Buzsaki, 2008), with recent data showing that NMDA antagonist induced gamma oscillations can be reversed by mGluR2/3 activation (Hiyoshi et al., 2014; Hikichi et al., 2015). In this study, the mixed mGluR2/3 agonist, LY379268 suppressed gamma and theta oscillations, an effect that was attenuated in the Han Wistar strain indicating a strong mGluR2 specific effect.

High frequency oscillations (130–160 Hz) occur in multiple regions including the cortex, hippocampus and nucleus accumbens (Buzsaki et al., 1983; Jones and Barth, 1999; Hunt et al., 2006) and are induced through psychotomimetic treatment (Hunt et al., 2006; Olszewski et al., 2013). Nonetheless, their physiological role is not fully understood. These current data suggest that modulation of mGluR2 or mGluR3 alone do not result in changes to HFO power (Fig. 5, Fig. 6).

### 4.5 Utility and limitations of Han Wistar and Wistar comparison

The Han Wistar rat strain has proved to be a useful model for studying mGluR2 versus mGluR3 receptor function in the absence of highly selective orthosteric ligands. The results obtained broadly agree with data for the knockout mouse model and also provide new insights through the recording of EEG data. Overall the mutation in the mGluR2 and resulting lack of functional protein expression did not result in baseline differences throughout any of these experiments. This may be a result of compensatory mechanisms that the Han Wistar rats have developed however they may also suggest that mGluR2 does not contribute a major role to neuromodulation under normal conditions. The lack of baseline differences suggests functional redundancy between mGluR2 and mGluR3, an effect suggested previously when comparing both single mGluR2^−/−^ and mGluR3^−/−^ knockout mice to double mGluR2/3^−/−^ mice in behavioural tasks (De Filippis et al., 2015). However, the differences in response to mGluR2/3 agonists and antagonists in these current data have revealed individual roles for each receptor.

Having a rat model to study mGluR2 versus mGluR3 functions is particularly useful for behavioural research where the rat is the more commonly used species. The larger size of the animal also makes the direct recording of brain function more feasible. However, the Han Wistar line was split from the non-Han Wistar more than 80 years ago (Wood et al., 2017). This will have resulted in wider genetic divergence between these rat strains than just the cys407* mutation within the Grm2 gene (Wood et al., 2017), meaning effects observed may also be influenced by other non-mGluR2 related differences. It is important to identify these caveats in this data given sleep processes are of a polygenic nature, with this comparison conducted in two different outbred lines, nonetheless, this spontaneous mutation within the Han Wistar rats has provided a novel avenue of analyzing the role of mGluR2 and mGluR3 in behaviour and processes such as sleep. In order to address this, heterozygote parents from the Han Wistar line could be used to provide litter-matched controls.

## 5 Conclusion

These data illustrate the significant contribution of mGluR2 in the antipsychotic-like, sleep and EEG effects of drugs acting at group II mGluRs through the use of Han Wistar rats that lack mGluR2 expression. We provide evidence for a role of mGluR3 activity in sleep-wake architecture and network oscillations using the novel mGluR2/3 antagonist LY3020371, with theta and gamma oscillations influenced by manipulations of mGluR2 or mGluR3 alone. Further distinguishing the subtype specific roles of these receptors will be critical for capitalizing on the vast potential of group II mGluR drugs, with the Han Wistar rats a novel tool for these investigations.
