# Supplementary material for: Investigating the role of mGluR2 versus mGluR3 in antipsychotic-like effects, sleep-wake architecture and network oscillatory activity using novel Han Wistar rats lacking mGluR2 expression
Source: Neuropharmacology. 2018 Sep 15;140:246–59. doi: 10.1016/j.neuropharm.2018.07.013 (PMC6137075; doi:10.1016/j.neuropharm.2018.07.013)
Supplement: Online data [file mmc1.docx]

**Supplementary Data**


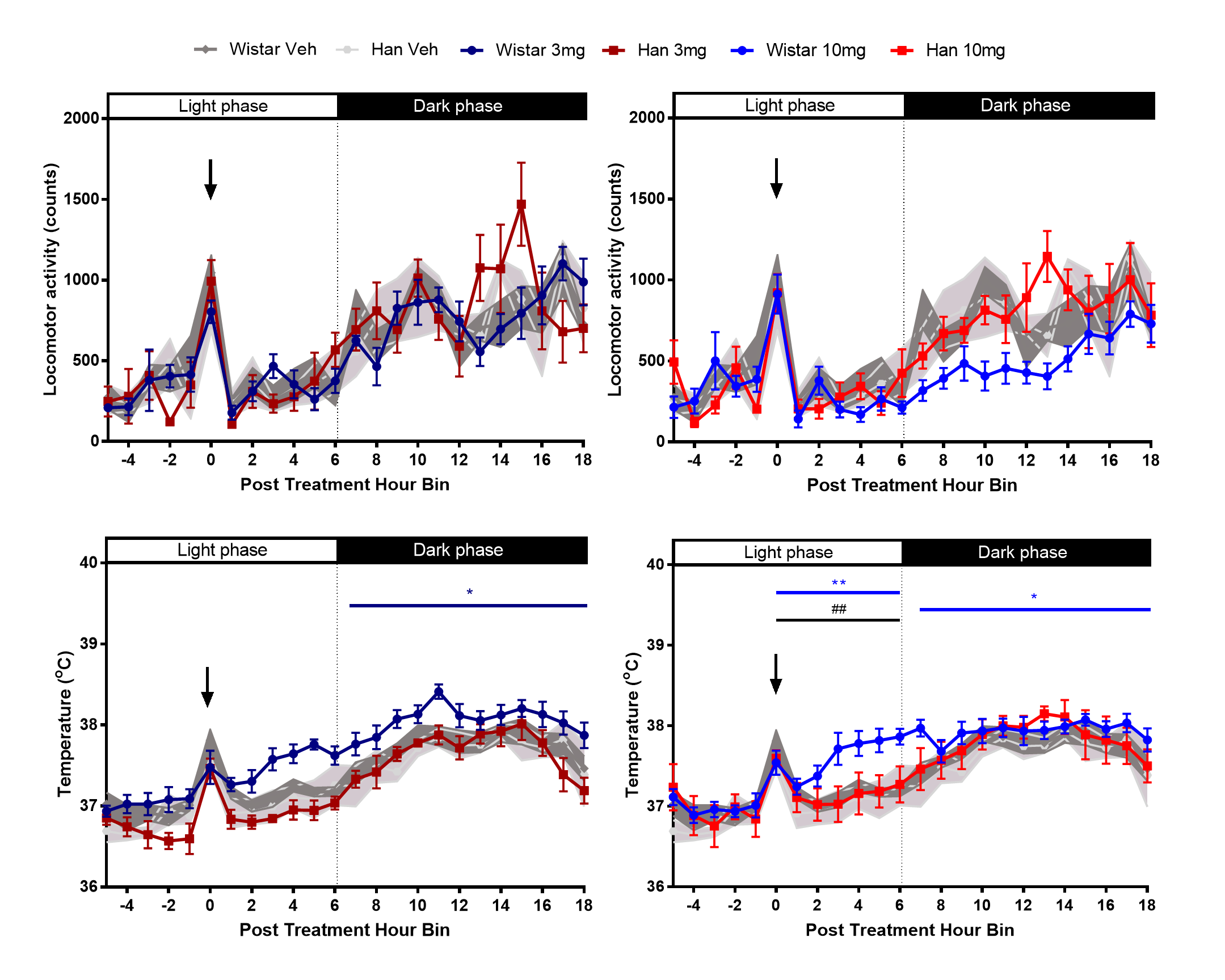


**Supplementary Figure 1. Effects of mGluR2/3 receptor agonist LY379268 (3, 10mg/kg) on locomotor activity and body temperature in Wistar and Han Wistar rats.** Mean locomotor activity counts and body temperature for each hour throughout the post treatment period are displayed as mean ± SEM. Significant treatment effects (*) and strain differences (#) are indicated as appropriate using significance values of p<0.05*, p<0.01** and p<0.001***.


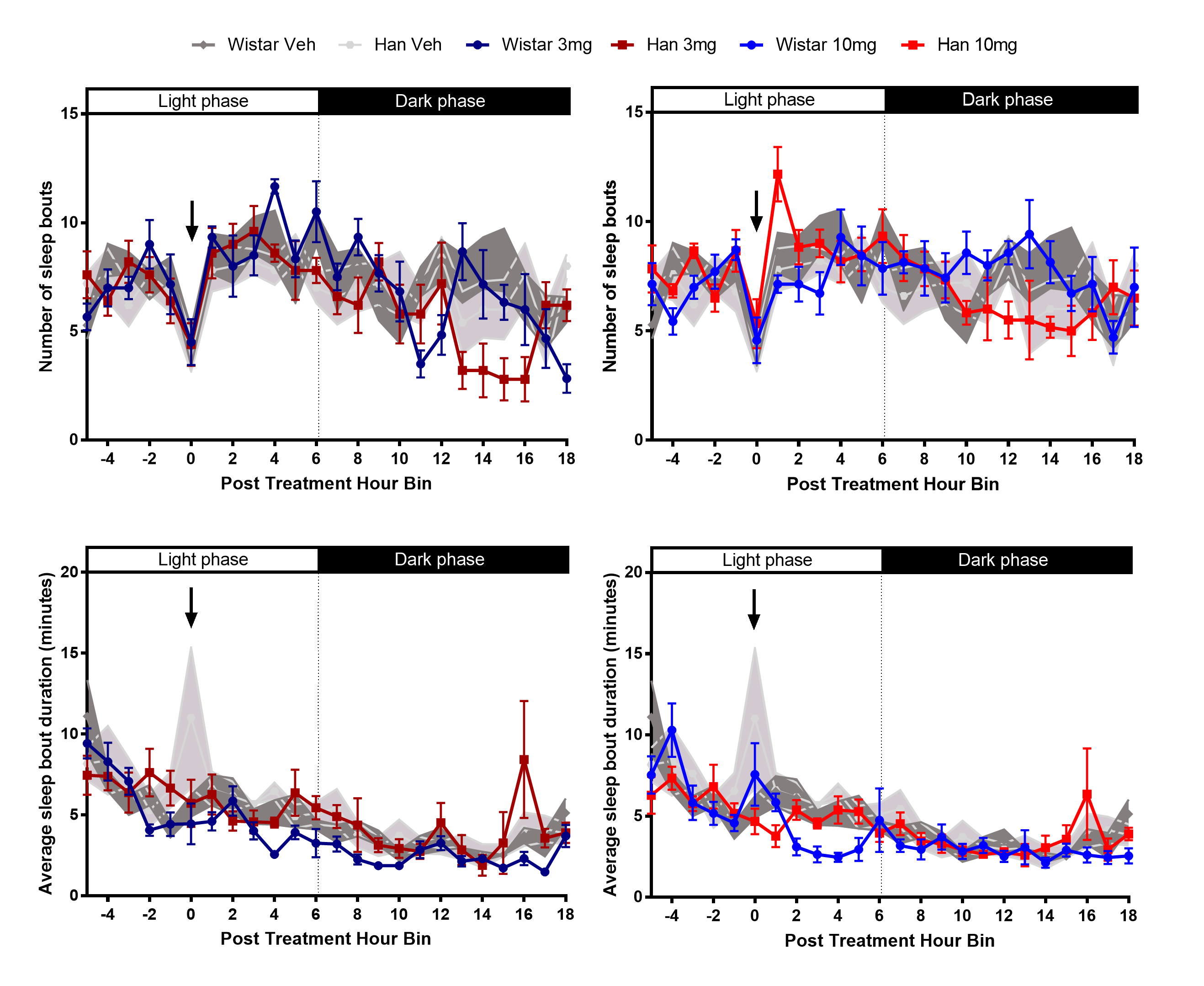


**Supplementary Figure 2. Effects of mGluR2/3 receptor agonist LY379268 (3, 10mg/kg) on sleep bout number and sleep bout duration in Wistar and Han Wistar rats.** The average number of sleep bouts and average sleep bout duration for each hour throughout the post treatment period are displayed as mean ± SEM.


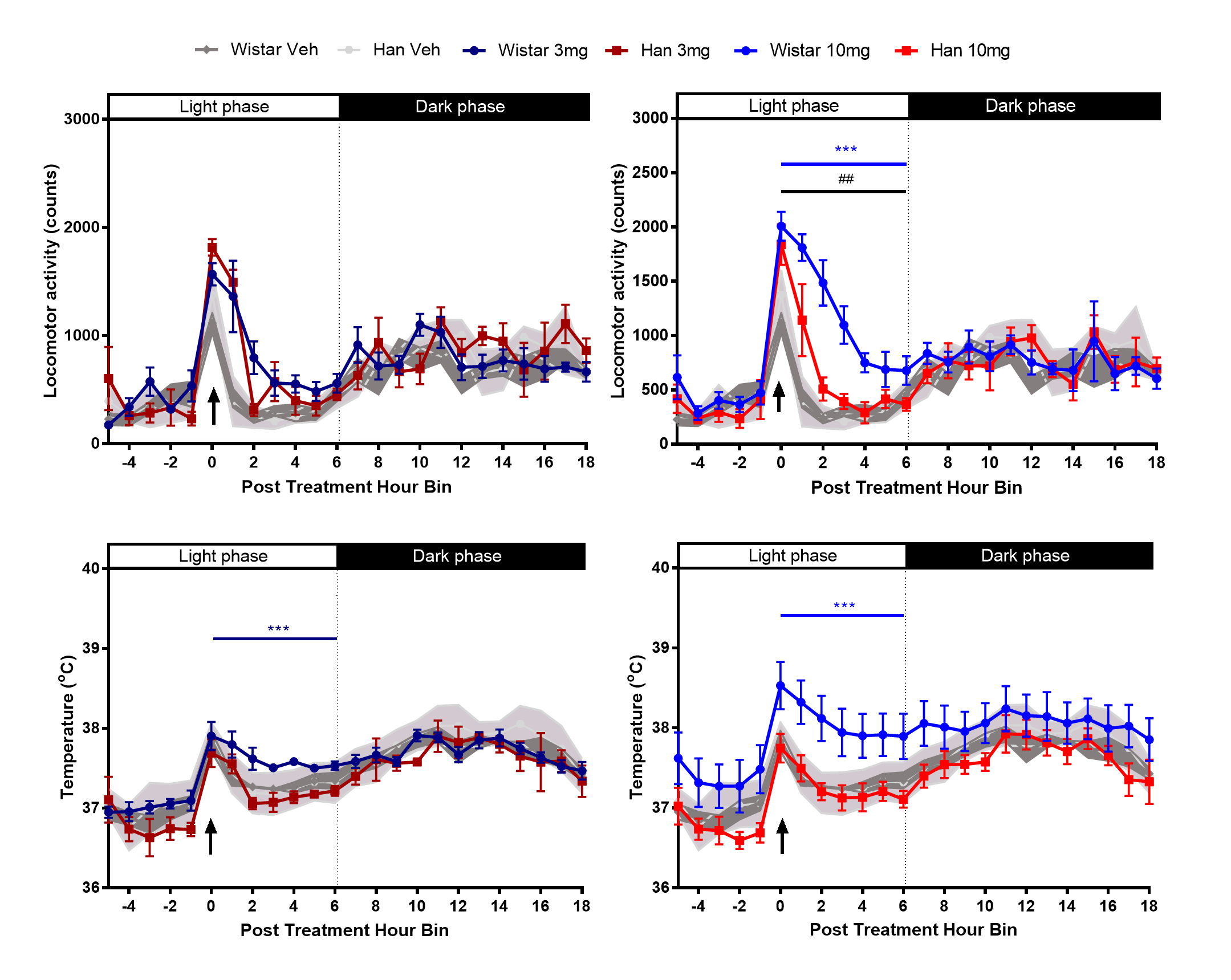


**Supplementary Figure 3. Effects of mGluR2/3 receptor agonist LY3020371 (3, 10mg/kg) on locomotor activity and body temperature in Wistar and Han Wistar rats.** Mean locomotor activity counts and body temperature for each hour throughout the post treatment period are displayed as mean ± SEM. Significant treatment effects (*) and strain differences (#) are indicated as appropriate using significance values of p<0.01** and p<0.001***.


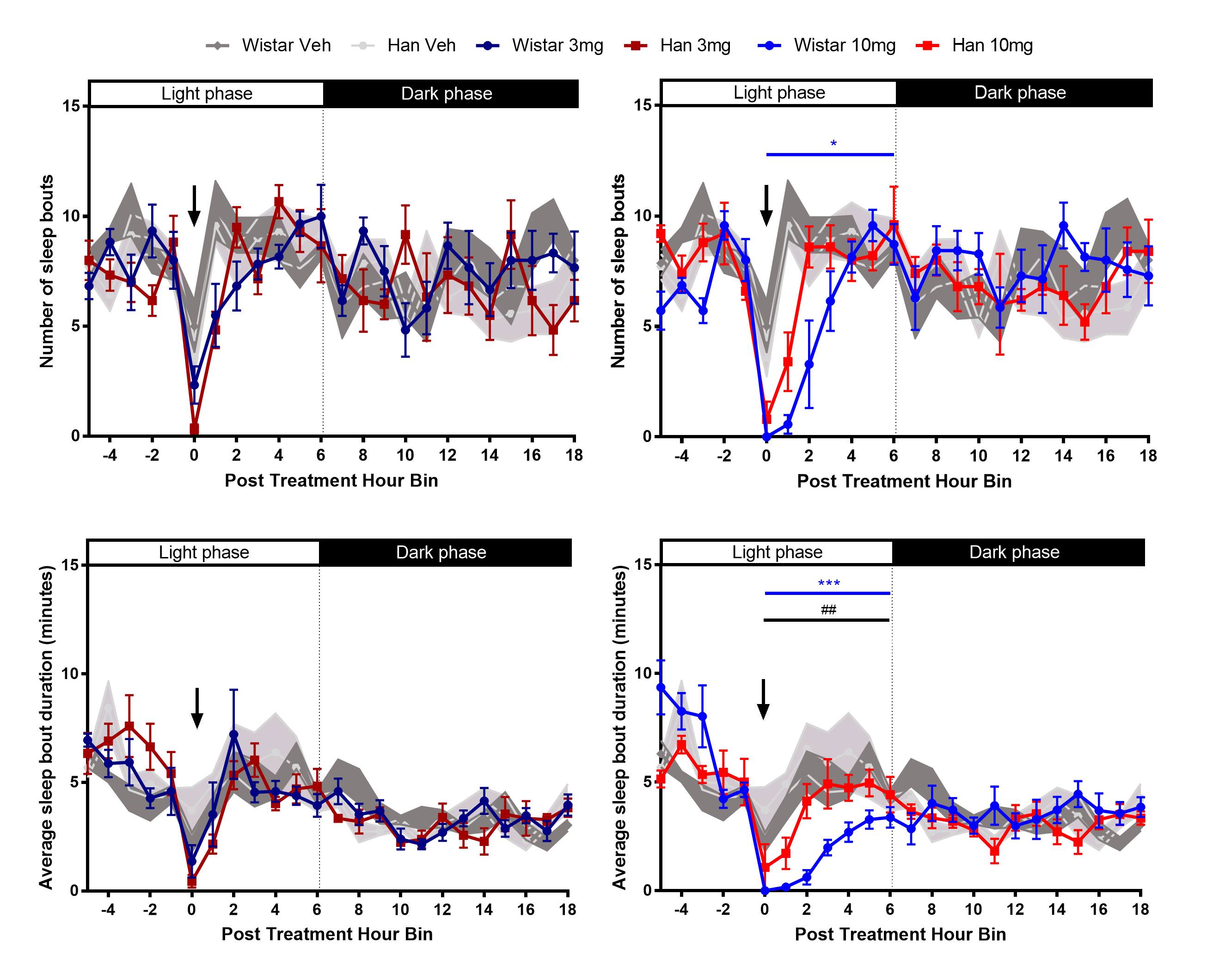


**Supplementary Figure 4. Effects of mGluR2/3 receptor antagonist LY3020371 (3, 10mg/kg) on sleep bout number and sleep bout duration in Wistar and Han Wistar rats.** The average number of sleep bouts and average sleep bout duration for each hour throughout the post treatment period are displayed as mean ± SEM. Significant treatment effects (*) and strain differences (#) are indicated as appropriate using significance values of p<0.05*, p<0.01** and p<0.001***.
